# A Comparison Between Rome III and Rome IV Criteria in Children with Chronic Abdominal Pain: A Prospective Observational Cohort Study

**DOI:** 10.5152/tjg.2022.21893

**Published:** 2022-11-01

**Authors:** Kaan Demirören, Bünyamin Güney, Muharrem Bostancı, Deniz Ekici

**Affiliations:** 1 Department of Pediatric Gastroenterology, Yüksek İhtisas Teaching Hospital, University of Health Sciences, Bursa, Turkey; 2 Department of Pediatrics, Yüksek İhtisas Teaching Hospital, University of Health Sciences, Bursa, Turkey

**Keywords:** Abdominal migraine, functional abdominal pain, functional dyspepsia, irritable bowel syndrome

## Abstract

**Background:**

The Rome IV includes a redefinition of functional gastrointestinal disorders and diagnostic criteria. The present study aimed to compare the Rome III and Rome IV classification results and to reveal their differences in children with chronic abdominal pain.

**Methods:**

The present study is a prospective observational cohort study. Three hundred forty-four children, who were admitted to the pediatric gastroenterology clinic, had abdominal pain for more than 2 months, and were not diagnosed with an organic disease, were included in our study.

**Results:**

In children with chronic abdominal pain, Rome IV criteria did not cause a change in the number of patients diagnosed with functional abdominal pain disorders according to Rome III (89.8% vs 89.2%, *P* >.05). Functional abdominal pain and functional abdominal pain syndrome were the most common diagnoses in Rome III and functional abdominal pain, not otherwise specified in Rome IV. When compared to Rome III, while the diagnosis of functional dyspepsia increased in Rome IV, irritable bowel syndrome decreased.

**Conclusion:**

In children with chronic abdominal pain, Rome IV criteria did not cause a change in the number of patients diagnosed with functional abdominal pain disorders according to Rome III, but it caused a diagnostic shift. It was seen that some of the children diagnosed with irritable bowel syndrome in Rome III shifted to functional dyspepsia diagnosis in Rome IV.

**Main Points**

- Rome IV criteria did not cause a change in the number of patients diagnosed with functional abdominal pain according to Rome III.
- The Rome IV criteria made it easier to diagnose functional abdominal pain.
- The diagnosis of functional dyspepsia increased in Rome IV according to Rome III.
- The diagnosis of irritable bowel syndrome decreased in Rome IV according to Rome III.

## Introduction

Chronic abdominal pain is an important problem with prolonged, intermittent, or persistent abdominal pain, which is widely used in pediatric practice, and is the cause of 2%-4% of all pediatric admissions.^1,2^ It is mostly functional, but doctors are still trying to find an organic cause, even if there is no evidence of an underlying organic disease. The worldwide pooled prevalence was reported for functional abdominal pain disorders (FAPDs) as 13.5%.^3^

Functional gastrointestinal disorders (FGIDs) are considered as morphologic and physiological abnormalities originated from dysmotility, visceral hypersensitivity due to changed mucosal and immune function, microbiota, central nervous system processing, and genetics.^4-8^ Functional gastrointestinal disorders are widely seen in pediatric populations of all ages and are linked to a decreased quality of life.^9-10^ The prevalence rates of pediatric FGIDs have been reported to be between 9.9% and 27.5% in children/adolescents.^10^

The absence of biomarkers or specific tests to diagnose FGIDs has led them to be diagnosed by symptom-based criteria. The Rome criteria are symptom-based guidelines, the foundations of which were laid in the 1990s, the third version was updated in 2006, and the fourth version was updated in 2016 and came into use.^4,11,12^

The current criteria used to diagnose FGIDs were published in 2016 and were named the Rome IV criteria.^13^ The Rome IV includes a redefinition of FGIDs and diagnostic criteria, with the newly recognized disorders, and some major changes in criteria for existing disorders.^14^ In the previous FGIDs’ criteria, “No evidence of an inflammatory, anatomic, metabolic, or neoplastic process that explains the subject’s symptoms” was a necessity. This criterion has been updated as “after appropriate medical evaluation, the symptoms cannot be attributed to another medical condition” in Rome IV.^13^ This change allows the clinician to perform whether a selective or no testing to support a positive diagnosis of an FGID. It is also shown that FGIDs can coexist with other medical conditions.^15^

The pediatric FGIDs criteria divide 2 groups according to ages: neonates and toddlers, and children and adolescents. Within the children and adolescents’ group of diagnoses, there are 3 diagnostic categories: disorders of nausea and vomiting, defecation disorders, and abdominal pain disorders, which are also the focus of our study.^13^

As for FAPDs in children and adolescents, the diagnosis title of “abdominal pain related functional gastrointestinal disorders” (AP-FGIDs) in Rome III has been updated as “functional abdominal pain disorders” (FAPDs) in Rome IV. The subtitles of “functional abdominal pain” and “functional abdominal pain syndrome” in Rome III have been replaced by the subtitle of “Functional abdominal pain not otherwise specified” (FAP-NOS) in Rome IV. Thus, FAP-NOS has started to be used as a term for patients who do not meet the irritable bowel syndrome (IBS), functional dyspepsia (FD), or abdominal migraine criteria.^15^

The aim of this study was to compare the Rome III and Rome IV classification results and to reveal their differences in children with chronic abdominal pain.

## Materials and Methods

### Patients

The present study is a prospective observational cohort study. Ethics committee approval numbered 2017-14/15 was obtained by the local Clinical Research Ethics Committee. Written consent was obtained from patients and/or their parents. The patients were evaluated with their parents. Patient information was kept confidential, and the study was conducted according to the Helsinki declaration.

### Including Criteria

Children and adolescent patients between the ages of 4 and 18 years who have been referred to the pediatric gastroenterology outpatient clinic and who have had abdominal pain for at least 2 months were evaluated by a pediatric gastroenterologist, and the patients who considered the diagnosis of FGID were included in the study. The prevalence of FAPDs in 4- to 18-years-old children is 10.4%.^16^ The population of 4- to 18-year-olds in the city where the study was performed is 433 000. According to these data, with 95% CI and 5% margin of error, the calculated sample size was 144 patients. In addition, approximately 600 patients admitted to our pediatric gastroenterology outpatient clinic in a month. About 40% of these are patients with chronic abdominal pain symptoms. Out of a total 7200 patients in a year, approximately 2880 patients have chronic abdominal pain. With 95% CI and 5% margin of error with these data, the sample size of the study was determined as 340 patients.

### Excluding Criteria

Medical evaluation included a medical history, reviewing past medical records, family and social history, system examination, and completion of a physical examination. The patients with alarm signs and/or symptoms^15^ (i.e., family history of inflammatory bowel disease, celiac disease, or peptic ulcer disease, persistent right upper or right lower quadrant pain, dysphagia, odynophagia, persistent vomiting, gastrointestinal blood loss, nocturnal diarrhea, arthritis, perirectal disease, involuntary weight loss, growth retardation, delayed puberty, unexplained fever) were evaluated with laboratory tests and endoscopy, if necessary. The patients with an organic pathology, chronic disease, or disability were excluded from the study.

### The Determination of Diagnostic Criteria

A questionnaire on pediatric gastrointestinal symptoms was reported in the literature for the diagnosis of Rome criteria.^17^ Due to the changing diagnostic criteria, the questionnaire shown in Table 1 was applied to all patients in the present study. This questionnaire was prepared by the patients’ parents and, if possible, from the patients themselves, together with their parents. According to the results of the questionnaire, the diagnoses were classified according to both the criteria of Rome III AP-FGIDs (FD, IBS, abdominal migraine [AM], functional abdominal pain [FAP], and functional abdominal pain syndrome [FAPS])^18,19^ and the criteria of Rome IV FAPDs (FD, IBS, AM, and FAP-NOS)^15^ by a single pediatric gastroenterologist.

### Statistical Analysis

Statistical analysis was performed using a statistical analysis package program (Statistical Package for the Social Sciences 20.0, IBM Corp.; Armonk, NY, USA). Firstly, descriptive analyses were made. Number and percentage values were given for grouped variables in descriptive analysis. The mean, standard deviation, minimum, and maximum values are given for other variables. McNemar test was applied for dependent-group categorical variables. *P* < .05 was considered statistically significant.

## Results

Of 2880 patients with chronic abdominal pain, 344 were included in the study. Of the patients, 185 (53.8%) were female and 159 (46.2%) were male. The mean age of the patients was 128.4 ± 54.4 months. The age ranges of the patients were as follows: 74 (21.6%) patients were 4-6 years old; 124 (36.3%) of the patients were 7-12 years old; and 144 (42.1%) of the patients were 13-18 years old. The age and sex distribution of the patients according to Rome III and Rome IV classifications are shown in Table 2.

**Figure 1.**
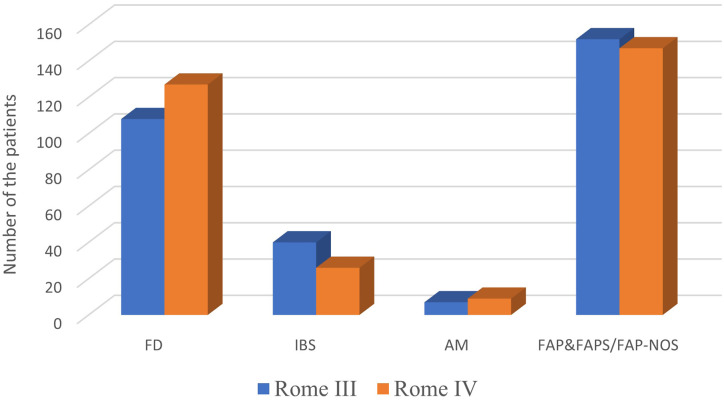
The frequency of the diagnoses of functional dyspepsia (FD), irritable bowel syndrome (IBS), abdominal migraine (AM), functional abdominal pain (FAP), functional abdominal pain syndrome (FAPS), and functional abdominal pain, not otherwise specified (FAP-NOS) according to Rome III and Rome IV criteria.

Of the 344 patients included in the study, 108 (31.4%) were diagnosed with FD according to Rome III, while 127 (36.9%) were diagnosed with FD according to Rome IV. This increase in the diagnosis of FD in Rome IV compared to Rome III was statistically significant (*P* < .05). The number of patients diagnosed with IBS was 40 (11.6%) in Rome III compared to 26 (7.6%) in Rome IV. This difference was also statistically significant (*P* < .05). While there were 152 (44.2%) patients diagnosed with FAP and FAPS in Rome III, there were 147 (42.7%) patients diagnosed with FAP-NOS in Rome IV (*P* > .05). Abdominal migraine was diagnosed in 7 (2%) cases in Rome III and in 9 (2.6%) cases in Rome IV (*P* > .05). The most common diagnoses were FAP and FAPS in Rome III, while FAP-NOS in Rome IV. When moving from Rome III to Rome IV, the diagnosis that increased the most was FD, and the diagnosis that decreased the most was IBS. Comparisons of the diagnoses according to Rome III and Rome IV classifications are seen in Table 3 and Figure 1.

## Discussion

Rome IV committee did not consider it necessary to perform endoscopy when diagnosing FD. However, they also stated that endoscopy cannot be avoided depending on local and social habits and approaches.^15^ The symptoms of organic diseases such as reflux esophagitis and eosinophilic esophagitis may be similar to the symptoms of FD.^20^ Therefore, we did not include patients with alarm symptoms in our study because we performed endoscopy even though Rome IV was not considered necessary. In the present study, the diagnosis of FD increased in Rome IV (Rome III, 31.4% vs Rome IV, 36.9%). Two subgroups for FD have been defined in Rome IV: epigastric pain syndrome and postprandial distress syndrome.^15^ In both subtypes, the diagnoses can be confused, as symptoms may be relieved after meals.^18,19^ Therefore, the diagnosis of FD was used as an umbrella diagnosis in this study. Other than that, the changes in Rome IV for FD are minimal.^20-22^

For IBS, the abdominal discomfort criterion was dropped from Rome IV. The criteria for “onset associated with a change with a change in frequency and form of stool” were changed as “a change in stool frequency and form.” At the same time, 2 out of 3 items related to defecation were required in Rome III, while one was deemed sufficient in Rome IV. We think this is a very appropriate change because when the patient and their parents were questioned, they had difficulty distinguishing these three items related to defecation from each other. In addition, in children with constipation, the pain that does not resolve with resolution of the constipation was added as a diagnostic criterion.^12,15,18^ In the present study, fewer patients were diagnosed with IBS according to Rome IV criteria (Rome III, 11.6% vs Rome IV, 7.6%). We believe that the most important reason for this decrease in the number of patients with IBS was the exclusion of abdominal discomfort in Rome IV and only the presence of pain. The term “abdominal discomfort” was dropped because it was both nonspecific and has different meanings in different languages.^22^ At the same time, “abdominal discomfort” was confused with FD symptoms such as postprandial fullness, abdominal bloating, postprandial nausea, or excessive belching. When it was understood that the complaint was not abdominal pain but a feeling of discomfort in the abdomen, the diagnosis of IBS was removed and its relationship with defecation became insignificant. After that, the focus was on FD, which was the first diagnosis to be considered. We think that this is the reason why the diagnosis of FD increased while the diagnosis of IBS decreased in our study.

Edwards et al^23^ in a similar study, compared Rome III and IV criteria in children with chronic abdominal pain and reported that the frequency of diagnosis in both FD and IBS (approximately 2 times), and also the overlap diagnosis of FD/IBS (3 times) increased in Rome IV. They suggested that the reason for this was that Rome IV approved the only substance related to the reduction of pain with defecation in IBS. However, they ignored the diagnoses of AM and FAP-NOS in their studies. We believe that all subtitles of “Functional abdominal pain disorders” should be evaluated together. If we ignored the subtitles diagnoses of AM, FAP&FAPS, and FAP-NOS in our study, there would have been a significant increase in the number of FD diagnoses. Indeed, Bai et al^24^ reported in a cross-sectional survey in adults that the diagnosis of IBS in Rome IV was approximately half of that of Rome III. They suggested like us that this was associated with the presence and absence of abdominal pain. In a large-scale multi-national study most recently published, the incidence of IBS was decreased in Rome IV compared to Rome III.^9^ As a matter of fact, with the removal of discomfort in the diagnosis of IBS and only pain remaining, Rome IV IBS patients were found to have higher gastrointestinal symptom severity and lower quality of life compared to Rome III IBS patients in another study where the diagnosis of IBS decreased in Rome IV.^25^

For AM in Rome IV, stereotypical pattern and symptoms in the individual patient was added as a diagnostic criterion, and the condition of the duration of symptoms was reduced from 12 months to 6 months.^12,15,18,19^ There was no difference in the number of patients diagnosed with AM in the present study (Rome III, 2% vs Rome IV, 2.6%). Since the presence of additional symptoms, such as headache, nausea, and photophobia, in the diagnosis of AM is more important than the duration of symptoms, it may be thought that shortening the duration of symptoms does not cause an increase in the number of diagnoses.

The condition of “insufficient criteria for IBS, FD, or AM” is required to diagnose FAP-NOS in Rome IV and FAP and FAPS in Rome III. Since the diagnosis of FAPS was completely excluded in Rome IV, additional symptoms such as loss of daily functioning, headache, and difficulty in sleeping, etc., were excluded from the diagnostic criteria as these can accompany other Rome diagnoses. The wording “that does not occur solely during physiologic events (e.g., eating, menses)” has been added.^12,15,18^ Therefore, there was no significant difference in the number of patients with Rome III (Rome III, 44.2%, Rome IV 42.7%).

We think that the most important diagnosis to be distinguished is FD when diagnosing FAP and FAPS in Rome III or FAP-NOS in Rome IV. Since FAP-NOS is not related to defecation, it can easily be differentiated from the diagnosis of IBS. However, it was difficult to decide whether these patients should be diagnosed with FD or FAP-NOS in the presence of non-epigastric abdominal pain together with postprandial fullness and/or early satiation. If these patients were evaluated as FD, the diagnosis of FD would increase while the number of FAP-NOS decreased in the present study, similar to other studies.^10,16^ As we understand, generalized abdominal pain should not be present when diagnosing FD according to Rome IV criteria. Because the phrase “The pain is not generalized or localized to other abdominal or chest regions” is included in “epigastric pain syndrome,” 1 of the 2 subtypes of FD. In the other subtype, “postprandial distress syndrome,” pain is not mentioned at all.^15^ Therefore, we preferred to diagnose FAP-NOS in patients with generalized abdominal pain, even with postprandial fullness and/or early satiety. This can be stated more clearly in the next Rome criteria.

In children with chronic abdominal pain, Rome IV criteria did not cause a change in the number of patients diagnosed with FGIB according to Rome III (89.8% vs 89.2%, *P* > .05). In the literature, the overall Rome IV prevalence rates were not changed meaningfully from Rome III in children and adolescents ages (4-18 years old),^10,16,26^ but a decrease has also been reported.^27^ While the most common diagnosis of AP-FGIDs were AM^10,26^ and IBS^16^ in Rome III, FD^10,16^ was the most common diagnosis of FAPDs in Rome IV. In 1 of 2 prevalence studies, it was reported that IBS diagnosis increased in Rome IV compared to Rome III,^10^ decreased in the other,^16^ and FD diagnosis increased in both studies. In the present study, FAP and FAPS were the most common diagnoses in Rome III and FAP-NOS in Rome IV. According to Rome III, while the diagnosis of FD increased in Rome IV, IBS decreased.

In conclusion, Rome IV criteria did not cause a change in the number of patients diagnosed with FGIB according to Rome III, whereas it caused a diagnostic shift. The present study demonstrated that some of the children diagnosed with IBS in Rome III shifted to FD diagnosis in Rome IV.

## Figures and Tables

**Table 1.** Questionnaire by Adapting the Pediatric Gastrointestinal Symptom Questionnaire for Rome III and IV Criteria

**Pediatric Gastrointestinal Symptom Questionnaire for Functional Abdominal Pain Disorders of Rome III and IV Criteria**
**Duration of abdominal pain/discomfort** • Pain: • Discomfort:
**Frequency of abdominal pain/discomfort** • Pain: • Discomfort:
**How long does it take when there is pain/discomfort** • Pain: • Discomfort:
**Severity of abdominal pain/discomfort** • Excruciating pain • Excruciating discomfort • Pain that limits daily activities • Discomfort that limits daily activities • Pain that does not interfere with daily activities • Discomfort that does not interfere with daily activities
**Healthy period between episodes of pain/discomfort** • Pain: >1 week or <1 week • Discomfort: >1 week or < 1 week
**Stereotypical pattern** • Yes • No
**Spread of abdominal pain/discomfort** • Generalized pain • Generalized discomfort • Periumbilical pain • Periumbilical discomfort • Epigastric pain • Epigastric discomfort
**Relationship with defecation** • Increase • Decrease • Unrelated
**Relationship with stool frequency and consistency** • Starting with a change in stool frequency • Starting with a change in stool consistency • Is there a change in stool frequency? • Is there a change in stool consistency?
**Early satiation** • Yes • No
**Postprandial fullness** • Yes • No
**Situation of pain/discomfort after constipation has resolved** • Pain/discomfort continues • Pain/discomfort decreases • Variable
**Conditions accompanying abdominal pain** • Anorexia • Nausea • Vomiting • Headache • Photophobia • Pallor

**Table 2.** Age and Sex Distribution of the Patients with Functional Gastrointestinal Disorders According to Rome III and Rome IV Classifications

FGIDs	Rome III			Rome IV		
	Female (n, %)	Male (n, %)	Age (Mean ± SD)	Female (n, %)	Male (n, %)	Age (Mean ± SD)
FD	69 (63.9)	39 (36.1)	154.5 ± 51.4	79 (62.2)	48 (37.8)	152.6 ± 52
IBS	14 (35)	26 (65)	125.5 ± 60	9 (34.6)	17 (65.4)	123.3 ± 63.5
AM	4 (57.1)	3 (42.9)	146.4 ± 48	5 (55.6)	4 (45.4)	136.5 ± 51.3
FAP & FAPS	83 (54.6)	69 (45.4)	110.1 ± 48.1	-	-	-
FAP-NOS	-	-	-	78 (53)	69 (47)	108.3 ± 47.1
Unclassified	15 (40.5)	22 (59.5)	126.8 ± 52.5	14 (40)	21 (60)	126.8 ± 51.6

FGIDs,** f**unctional gastrointestinal disorders; FD, functional dyspepsia; IBS, irritable bowel syndrome; AM, abdominal migraine; FAP, functional abdominal pain; FAPS, functional abdominal pain syndrome; FAP-NOS, functional abdominal pain not otherwise specified.

**Table 3.** The Comparison of the Diagnoses of the Patients with Functional Gastrointestinal Disorders According to Rome III and Rome IV Classifications

FGIDs	Rome III, n (%)	Rome IV, n (%)
FD*	108 (31.4)	127 (36.9)
IBS*	40 (11.6)	26 (7.6)
AM	7 (2)	9 (2.6)
FAP & FAPS	152 (44.2)	-
FAP-NOS	-	147 (42.7)
Unclassified cases	37 (10.8)	35 (10.2)

FGIDs, functional gastrointestinal disorders; FD, functional dyspepsia; IBS, irritable bowel syndrome; AM, abdominal migraine; FAP, functional abdominal pain; FAPS, functional abdominal pain syndrome; FAp-NOS, functional abdominal pain not otherwise specified.

*P < .001.
